# Rupture utérine spontanée sur léiomyosarcome utérin

**DOI:** 10.11604/pamj.2016.24.86.8849

**Published:** 2016-05-27

**Authors:** Khouloud Boussouni, Meryem Benoulaid, Rachida Dafiri

**Affiliations:** 1 Service de Radiologie de l'Hôpital d'Enfants, Maternité Souissi, Centre Hospitalier Ibn Sina, 40000 Rabat, Maroc

**Keywords:** Léiomyosarcome, hémopneumopéritoine, rupture utérine, TDM, leiomyosarcoma, hemo-pneumoperitoneum, uterine rupture, CT scan

## Abstract

Les sarcomes utérins sont des cancers rares caractérisés par leur polymorphisme clinique et histologique. Ce sont des tumeurs malignes de mauvais pronostic. Ils se révèlent, généralement, par des symptômes non spécifiques à type de douleurs pelviennes, métrorragies. Un tableau d'abdomen aigu avec hémopneumopéritoine est une présentation rare. Nous rapportons le cas d'un léiomyosarcome utérin qui s'est manifesté par une péritonite aigue généralisée inaugurale chez une femme de 43 ans. Le diagnostic de perforation utérine sur tumeur maligne a été suspecté par l'imagerie préopératoire en urgence (échographie et TDM) et confirmé histologiquement.

## Abstract (en)

Uterine sarcomas are rare cancers characterized by clinical and histological polymorphism. They are malignant tumors of poor prognosis. Inaugural manifestation of acute abdomen with hemo-pneumoperitoneum and spontaneous uterine rupture remains exceptional. The authors report the case of a uterine leiomyosarcoma manifesting itself as an inaugural generalized acute peritonitis in a 43 year old woman. Uterine rupture due to malignant tumor was suspected during urgent preoperative imaging (ultrasound and CT scan) and confirmed histologically.

## Introduction

Les sarcomes utérins sont des cancers rares caractérisés par leur polymorphisme clinique et histologique. Ce sont des tumeurs malignes de mauvais pronostic. Ils se révèlent, généralement, par des symptômes non spécifiques à type de douleurs pelviennes, métrorragies. Un tableau d'abdomen aigu avec hémopneumopéritoine est une présentation rare. Nous rapportons le cas d'un léiomyosarcome utérin qui s'est manifesté par une péritonite aigue généralisée inaugurale chez une femme de 43 ans. Le diagnostic de perforation utérine sur tumeur maligne a été suspecté par l'imagerie préopératoire en urgence (échographie et TDM) et confirmé histologiquement.

## Patient et observation

**Figure 1:**
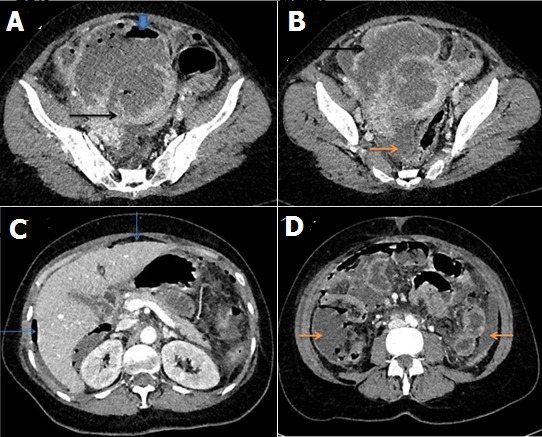
TDM abdomino-pelvienne en coupes axiales après injection du produit de contraste iodé au temps portal montrant un volumineux processus lésionnel à point de départ utérin (A,B), de densité tissulaire rehaussé de façon hétérogène après injection du produit de contraste délimitant de larges zones liquidiennes de nécrose et des bulles d'air. On note également l’épanchement liquidien et hématique intra péritonél (l'hémopéritoine) ainsi que le pneumopéritoine témoignant d'une péritonite par rupture utérine (C,D)

Il s'agit d'une patiente âgée de 43 ans, sans ATCD pathologiques particuliers nulligeste, en période de péri -ménopause, qui consulte en urgence pour des métrorragies minimes et douleurs pelviennes aigues. L'examen clinique retrouve une patiente en mauvais état général, présentant des signes de choc, une sensibilité abdominale diffuse avec défense généralisée.l'examen gynécologique met en évidence un col fermé sans masse latéro-utérine associée. Le bilan biologique révèle une hyperleucocytose à 15000elem/mm^3^, un syndrome inflammatoire avec une CRP à 62mg/l, une anémie hypochrome microcytaire à 11g/dl. Le diagnostic suspecté cliniquement est celui de GEU. L’échographie pelvienne réalisée par voie sus pubienne complétée par voie endovaginale retrouve un utérus augmenté de taille mesurant environ 120 x 80 x 60mm, siège d'un processus lésionnel hétérogène renfermant de multiples images aériques responsables d'artefacts gênant l'exploration. Présence d'un épanchement péritonéal liquidien finement échogène de moyenne abondance. Aucune image annéxielle en faveur d´une GEU n´a été individualisée. Le taux de BHCG plasmatique est revenu négatif. Un complément TDM abdomino pelvienne avant et après injection du produit de contraste iodée (au temps portal) a été réalisé (Figure 1). Il a confirmé la présence d'un processus lésionnel tumoral d'origine utérine tissulaire rehaussé de façon hétérogène après injection renfermant une composante liquidienne de nécrose et des bulles d'air (A,B). Il mesurait 58x40x35mm. Il existait également un pneumopéritoine avec un épanchement péritonéal de densité élevée (42UH) en faveur d'un hémopneumopéritoine (C, D). On a conclut à un processus tumoral utérin d'allure maligne compliqué d'une rupture utérine avec hémopneumopéritoine. La patiente a été opérée en urgence. L'exploration chirurgicale a retrouvé une volumineuse tumeur utérine nécrosée adhérente au péritoine pariétal sans extension macroscopique évidente à la paroi pelvienne ni aux parois digestives de la charnière rectosigmoidienne. Une hystérectomie totale avec annexectomie bilatérale a été réalisée. L'examen histologique de la pièce opératoire avec étude immunohistochimique est en faveur d'un léiomyosarcome utérin avec extension au péritoine pariétal. La patiente a été adressée en oncologie pour chimiothérapie et radiothérapie adjuvantes.

## Discussion

Les sarcomes utérins sont des tumeurs rares d'origine mésenchymateuse qui représentent entre 2 et 6% de l'ensemble des cancers utérins. Ce sont des cancers de mauvais pronostic puisque le taux de survie à 5ans varie de 10 à 60% selon la qualité d'exérèse initiale et le type histologique. Dans 70% des cas, ces tumeurs touchent surtout les femmes en post-ménopause [1]. Les manifestations cliniques des sarcomes utérins ne sont pas spécifiques et peuvent mimer des pathologies utérines bénignes en particulier celles des fibromes utérins. Ils se révèlent le plus souvent par des ménométrorragies et des douleurs pelviennes [2]. La rupture utérine sur sarcome utérin avec hémopéritoine spontané, comme le cas de notre observation, reste une présentation inhabituelle. La rupture utérine avec hémopéritoine est décrite surtout pour les léiomyomes. Environ 100 cas ont été rapportés. Le mécanisme évoqué est la rupture spontanée de veines superficielles ou la torsion d'un myome pédiculé [3]. Il existe très peu de cas décrits dans la littérature de sarcome utérin avec un tableau clinique inaugural menaçant le pronostic vital comme la rupture utérine ou l’état de choc hypovolémie. De Roy et Wiegerinck ont rapporté le cas d'une volumineuse tumeur utérine sarcomateuse à développement rapide avec hémopéritoine chez une fillette de 15 ans. Ils ont conclu que le mécanisme de l'hémorragie était lié aux connexions vasculaires de la tumeur plutôt qu’à la rupture utérine [4]. Une revue de la littérature actuelle retrouve seulement deux autres cas d'hémopéritoine secondaire à la rupture de leiomyosarcome utérins. Le mécanisme le plus vraisemblable à l'origine de rupture spontanée de léiomyosarcome est la nécrose tumorale [5, 6]. L'imagerie est d'une grande aide pour le diagnostic préopératoire des sarcomes utérins. Elle permet d'identifier les éléments sémiologiques de malignité permettant ainsi de codifier la thérapeutique. En effet 0,2 à 0,7% des tumeurs opérées avec le diagnostic préopératoire de fibromes se révèlent être des sarcomes [7]. L’échographie est l'examen de 1re intention réalisé en urgence devant une symptomatologie pelvienne aigue. En cas de leiomyosarcome compliqué de rupture utérine et d'hémopéritoine, elle détecte facilement la présence d’épanchement liquidien dans les différents récessus habituels du péritoine. L'hémopéritoine apparaît comme un épanchement hétérogène au niveau des zones déclives avec un aspect échogène de sédimentation au sein des composantes liquidiennes pures. Elle permet aussi la visualisation d'une volumineuse masse utérine tissulaire hétérogène largement nécrosée [8].

Cependant l’échographie reste insuffisante pour différencier de façon satisfaisante un myome remanié d'un sarcome sur les seuls critères morphologiques. L’échographie couplée au doppler couleur peut aider. La mesure des index de résistance a montré une différence significative entre les fibromes (index de résistance 0,59 +/- 0,01) et les sarcomes (index de résistance 0,41 +/- 0,06) [9]. Le scanner est aussi un examen souvent réalisé dans le contexte de l'urgence. En cas de leiomyosarcome compliqué de rupture utérine, il permet facilement de poser le diagnostic d'hémopéritoine devant un épanchement majoritairement liquidien avec des valeurs de densité mesurées entre 0 et 20 UH associé à une composante hématique correspondant à du sang coagulé (de densité spontanée élevée entre 40 et 70 UH) visible dans les récessus péritonéaux déclives [8]. La TDM peut aider à rattacher un volumineux processus tumoral pelvien à son origine utérine. Mais elle n'est pas sensible pour distinguer un léiomyosarcome d'un léiomyome en nécrobiose [10]. L'IRM pelvienne, réalisée en seconde intention permet de faire le bilan d'extension d'une lésion maligne histologiquement prouvée, de rattacher une masse volumineuse à son origine utérine et enfin de mieux caractériser les anomalies détectées à l’échographie. Cependant, l'IRM ne permet pas de distinguer de façon très fiable une lésion utérine bénigne d'une lésion maligne myométriale [11]. L'aspect en IRM des sarcomes utérins n'est pas très spécifique. Ils se présentent sous forme de lésions en iso ou hyposignal T1 (par rapport au signal du myomètre) avec possibles zones hémorragiques. En T2, ils ont un signal hétérogène avec des plages en hypersignal T2. Ils se rehaussent au temps artériel de façon intense et hétérogène après injection de produit de contraste. Cette prise de contraste est intense supérieure à celle du myomètre. L'existence de plages nécrotiques est un argument très en faveur de l'origine maligne sarcomateuse. En revanche, le problème de diagnostic différentiel se pose avec les fibromes remaniés en particulier les fibromes œdémateux ou en dégénérescence hyaline qui peuvent avoir un hypersignal T2 ou encore les fibromes compliqués de nécrobiose et qui se rehaussent de façon hétérogène après injection de gadolinuim [11]. C'est dans ces cas ou on note l'intérêt des séquences complémentaires notamment l'IRM de diffusion avec calcul de l'ADC. Plusieurs études ont analysé l'apport de la mesure de l'ADC dans la distinction entre sarcome et léiomyome. Tamai. K et al ont démontré qu'il existe une différence significative entre l'A DC des sarcomes et l'ADC des myomes avec une valeur seuil à 1,7, à l'exception de chevauchement en cas de myomes non remaniés mais en franc hypersignal T2 et les myomes hypercellulaires [12]. Le diagnostic de certitude des sarcomes utérins est histologique et se fait souvent en post-opératoire [1, 2]. Le traitement des sarcomes utérins est chirurgical. Il comprend l'hystérectomie totale avec annexectomie bilatérale en cas de tumeur limitées au corps utérin.l'indication du traitement adjuvant reste discutée [13]. Le pronostic des sarcomes utérins demeure péjoratif. La probabilité de survie à 5 ans est estimée à 30%, tout stade confondu [2].

## Conclusion

Les sarcomes utérins sont des cancers rares. Leur présentation par un tableau d´abdomen aigu inaugural avec hémopneumopéritoine et rupture utérine reste exceptionnelle. Il faut néanmoins y penser et inclure cette pathologie parmi la gamme diagnostiques des urgences pelviennes d´origine gynécologique. L'imagerie, notamment le scanner réalisé en urgence, doit orienter le diagnostic préopératoire de ces formes compliquées.
